# Predominantly Terrestrial Foraging and Reproductive Gains From a High Trophic Level Diet in Roof‐Nesting Herring Gulls (*Larus argentatus*)

**DOI:** 10.1002/ece3.72307

**Published:** 2025-10-10

**Authors:** Simon F. Allen, Richard Inger, Paige E. Petts, Jody M. Affleck, Katie Bennett, Tom W. Davies, Ben D. Haden, Luke C. Hughes, Neeltje J. Boogert, Chris Mitchell, Eva Jimenez‐Guri, Jonathan D. Blount

**Affiliations:** ^1^ Centre for Ecology & Conservation, Faculty of Environment, Science & Economy University of Exeter, Penryn Campus Penryn Cornwall UK

**Keywords:** breeding success, foraging choices, gull, stable isotopes, trophic level, urbanisation

## Abstract

Wild animal species often use human‐modified environments for foraging and reproduction, but this may require dietary diversification with fitness consequences. The extent to which colonising species successfully exploit such habitats is poorly understood. We used stable isotope analysis of egg yolk to investigate the association between foraging choices and reproductive success in 102 female herring gulls ( *Larus argentatus* ) over 3 years in a roof‐nesting, pericoastal breeding colony. Stable isotopes of egg yolk predominantly reflect maternal diet during egg production. We measured δ^13^C as an indicator of foraging habitat, and δ^15^N as an indicator of trophic level. We predicted diverse foraging choices across marine, terrestrial and urban environments due to gulls' generalist foraging strategy and the variety of nearby foraging opportunities. We also predicted higher reproductive success associated with marine feeding compared to terrestrial feeding or feeding on human food and refuse, because marine food has historically been gulls' natural food type and has been previously associated with greater reproductive success. Surprisingly, δ^13^C values indicated predominantly terrestrial foraging for egg production. Egg mass increased significantly with lower δ^13^C, indicative of more terrestrial feeding. These findings may reflect availability of habitats and foods nearby or indicate adaptive dietary choices. Fledging success increased significantly with elevated δ^15^N, indicating that mothers feeding at higher trophic levels before laying produced higher quality eggs and/or had superior offspring‐rearing capacity. A high trophic level maternal diet may nutritionally benefit offspring or improve parental condition. Egg stable isotope ratios of δ^13^C and δ^15^N were highly repeatable within clutches, enabling us to predict stable isotope values of unsampled eggs from sampled sibling eggs. Our results highlight high usage of terrestrial foods for egg production, whereas marine and anthropogenic feeding were rare. The reasons for this preference warrant further investigation to advance understanding of species that use human‐modified environments.

## Graphical abstract

We used stable isotope analysis to investigate the association between foraging choices and reproductive success in roof‐nesting herring gulls. Surprisingly, δ^13^C values indicated predominantly terrestrial foraging, while fledging success increased with elevated δ^15^N, indicating that mothers feeding at higher trophic levels produced higher quality eggs and/or had superior offspring‐rearing capacity. Our results highlight high usage of terrestrial foods for egg production; the reasons for this preference warrant further investigation to advance understanding of species that utilise human‐modified environments.

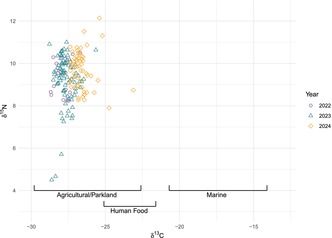

## 1 Introduction

Urbanisation is a leading source of habitat degradation, biodiversity loss and environmental change (Grimm et al. 2008; Piano et al. 2020; Dri et al. 2021). It brings drastic changes, including habitat loss (Beninde et al. 2015; Fattorini et al. 2018), substitution of natural foods with anthropogenic foods (Soto‐Calderón et al. 2016; El‐Sabaawi 2018; Gámez et al. 2022) and increased anthropogenic disturbance (Reijnen et al. 1997; Fernández‐Juricic and Tellería 2000; Markovchick‐Nicholls et al. 2008). Many species cannot compensate for urbanisation, whereas others adapt successfully (Blair 1996; McKinney 2006; Johnson and Munshi‐South 2017; Castillo‐Contreras et al. 2021). Some show improved body condition and reproductive success due to anthropogenic foods, reduced parasite exposure and lowered interspecific competition (Hegglin et al. 2007; French et al. 2008; Stout et al. 2010). For others, urban living becomes an ‘ecological trap’ (Dwernychuk and Boag 1972; Gates and Gysel 1978), with detrimental health impacts (Strasser and Heath 2013; Becker and Hall 2014; Soto‐Calderón et al. 2016; Murray et al. 2019).

Gulls have historically been considered generalist predators of primarily marine and intertidal prey (Spaans 1971; Pierotti and Annett 1987, 1991), but increasingly inhabit and feed in urban environments. In the United Kingdom, an estimated 60%–70% of herring gulls ( *Larus argentatus* ) nest on rooftops (Burnell 2021). Some gull populations now rely on anthropogenic food (Pons and Migot 1995; Steigerwald et al. 2015; Gyimesi et al. 2016; Goumas et al. 2020). Although anthropogenic food is convenient (Egunez et al. 2018), it can impact breeding success and body condition when prioritised over marine and intertidal diets (Pierotti and Annett 1991; Annett and Pierotti 1999; Ronconi et al. 2014; O'Hanlon et al. 2017). This may be because marine food provides a more suitable balance of nutrients required by gulls, especially micro‐nutrients crucial for breeding gulls and their chicks, such as calcium or omega‐3 fatty acids (Pierotti and Annett 1991; Bolton et al. 1992; Bukacińska et al. 1996; O'Hanlon et al. 2017; De Faria et al. 2021). In the UK, herring gulls are Red‐Listed, with internationally important populations (Stanbury et al. 2024). Cliff‐nesting populations have declined by 38% since 2002, but whether increasing urban populations compensate for this is unclear (Burnell 2021), making it crucial to understand factors influencing urban herring gull breeding success. As a dietary generalist showing individual specialisation (McCleery and Sibly 1986), herring gulls are ideal models for studying urbanisation effects on reproduction.

Stable isotopes are an established proxy for diet in ecological studies (reviewed by Peterson and Fry 1987), providing information about trophic level, foraging habitat and diet composition (Inger and Bearhop 2008). Stable isotope ratios (SIRs) in consumers' tissues reflect diet during tissue synthesis, providing a ‘snapshot’ of diet during a defined period based on tissue turnover rates (Hobson and Clark 1992). Previous stable isotope analyses focused on tissue samples such as blood and feathers (Ronconi et al. 2014; O'Hanlon et al. 2017; De Faria et al. 2021), gaining insights into parental diet during breeding, but few have sampled eggs (though see Hobson et al. 1997, 2000). Because egg nutrients originate in maternal diet and body storage, SIRs in eggs reflect maternal diet (Hobson 1995). Herring gulls and lesser black‐backed gulls ( *Larus fuscus* ) use endogenous and exogenous nutrients for egg formation, depending on food availability, with a significant proportion of these nutrients coming from exogenous sources (Houston et al. 1983; Hario et al. 1991; Hobson et al. 1997, 2000; Whiteman et al. 2021). This includes courtship feeding by males, which contributes to laying females' diets (Norstrom et al. 1986; Hario et al. 1991). Supplementation studies show that maternal diet components during egg formation, including proteins, carotenoids and vitamin E, influence egg quality and breeding success (Bolton 1991; Blount et al. 2002, 2004; Parolini et al. 2015). Herring gull eggs form 11–13 days before laying (Roudybush et al. 1979). Thus, SIRs in herring gull egg yolk are representative of maternal diet during this short, crucial period.

We used stable isotope analysis to investigate the diet of roof‐nesting herring gulls during egg formation and associations with breeding success. Nests were followed during laying in 2022–2024 and continuously from laying through fledging in 2023 and 2024. For a subset of nests, we removed whole clutches to determine consistency in SIRs across eggs within clutches. We predicted SIRs would not differ significantly over the laying sequence because they reflect maternal diet during a relatively short period of egg formation, when we would not expect huge diversity in individual foraging habitat selection. For all other sampled nests, one egg was removed from each clutch and replaced with a dummy egg, while remaining eggs were left to develop. The study population is < 2 km from the coast, with diverse coastal and intertidal habitat types within 10 km and surrounded by agricultural fields, mostly arable or permanent pasture and parkland. Because herring gulls show individual foraging specialisation, we predicted a variety of marine, terrestrial and anthropogenic foraging across these available habitat types. We also predicted marine feeding would be associated with higher reproductive success than anthropogenic or terrestrial feeding, as observed in other studies (Pierotti and Annett 1991; Annett and Pierotti 1999; O'Hanlon et al. 2017), because marine and intertidal resources are historically gulls' primary food source and are hypothesised to best meet breeding gulls' energy and nutrient requirements (Pierotti and Annett 1991; Bolton et al. 1992; Bukacińska et al. 1996).

## 2 Methods

### 2.1 Study Site

Fieldwork was conducted at the University of Exeter's Penryn Campus (Cornwall, UK: 50.170814° N, −5.1255223° W). The roofs of the campus buildings hold up to 100 breeding pairs of herring gulls and are accessible for population monitoring, being surrounded by safety barriers or equipped with fall‐arrest anchors for safe working at height using ropes and harnesses.

### 2.2 Sample and Data Collection

The study was carried out in 2022, 2023 and 2024. From early April, roofs were monitored daily for nests, and once laying commenced, all eggs were recorded and weighed using a portable electronic balance (±0.01 g; Shenzhen ACCT Electronic Co. Ltd, China). Each egg was marked A, B or C with a chinagraph pencil to denote the laying sequence. In 2022, we collected three full clutches and four partial clutches but did not monitor nests for the rest of the breeding season. In 2023, we collected seven whole clutches (five 3‐egg clutches and ten 2‐egg clutches). For other nests studied, we collected 1 randomly selected egg per clutch. There were 21 unsampled nests across 2023 and 2024, either because the nest was found after laying had finished, or because only 1 egg was present. Totals of eggs sampled and monitored are summarised in Table S1 and Figure S1.

In 2023 and 2024, nests were monitored daily. After hatching, chicks were weighed with an electronic balance (see above). For 112 chicks, a blood sample (< 0.1% body mass, as determined on the day of sampling) was taken from the tarsometatarsal vein by capillary tube on day 3 (±1 day) for molecular sexing, following veterinary advice and UK Home Office regulations. After sampling, gentle pressure was applied to the puncture site using cotton wool for at least 1 min to encourage clotting and prevent subcutaneous bleeding, which can lead to unintended fluid loss in small birds (Voss et al. 2010). Hatchlings were individually marked with coloured nail polish under the bill, then fitted with British Trust for Ornithology (BTO) numbered rings at 11–13 days of age. Nests were monitored until Day 35 when chicks were deemed to have fledged. To prevent straying, a 0.5 m high, approximately 2.5 m radius chicken wire fence was placed around each nest before chicks reached Day 3. Gulls habituate to such enclosures, which do not impact parental nesting behaviour (Bolton 1991; Nager et al. 2000; Kavelaars et al. 2019).

### 2.3 Stable Isotope Analysis

Yolk samples were dried in Eppendorf tubes for 48 h at 100°C in a drying oven. Lipids are depleted in ^13^C relative to proteins and contain minimal nitrogen; therefore, removing lipids means carbon isotope ratios better reflect diet, with negligible effects on δ^15^N values (Ingram et al. 2007; Ricca et al. 2007). To remove lipids, 25 mg of dried egg yolk were transferred to a tube with 1 mL of cyclohexane. Tubes were lightly agitated for 1 h using a benchtop shaker, then centrifuged for 5 min at 3000 *g*. The supernatant was removed, samples were dried again as described, then ground into fine powder by mortar and pestle. 0.8 mg (±10%) were transferred into a tin capsule for analysis. SIRs of carbon (δ^13^C) and nitrogen (δ^15^N) were measured using a Sercon Integra 2 continuous flow stable isotope mass spectrometer. SIRs are reported as δ‐values in ‰, according to: δ X = [(*R*_sample_/*R*_standard_) − 1] × 1000, where X is ^13^C or ^15^N and R is the corresponding ratio ^13^C/^12^C or ^15^N/^14^N, and *R*_standard_ is the ratio of the international references PDB for carbon and AIR for nitrogen. Replicate analyses of internal lab reference material (bovine liver; δ^13^C −28.61, δ^15^N 6.32 and Alanine; δ^13^C −19.62, δ^15^N −1.85) were used to correct for instrument drift and determine sample δ^13^C and δ^15^N values. %C and %N were determined using the beam area relative to the bovine liver standard (%C 47.24, %N 9.31). Across runs, the mean precision was 0.08 for δ^13^C and 0.165 for δ^15^N (two standard deviations). In total, we measured δ^13^C and δ^15^N for 164 egg yolks: 18 in 2022, 89 in 2023 and 57 in 2024. To distinguish between natural terrestrial, anthropogenic and marine stable isotope signatures, we compared our δ^13^C values to those from a range of other studies (Peterson and Fry 1987; Hobson et al. 1994; O'Connell and Hedges 1999; Knoff et al. 2002; Hopkins and Ferguson 2012; Ronconi et al. 2014; Shlepr et al. 2021; Fletcher et al. 2025). The diet‐tissue fractionation factor for δ^13^C in lipid‐free yolk is negligible (Hobson 1995), aiding in comparison between studies.

### 2.4 Molecular Sexing

Blood samples were separated into plasma and red blood cells by centrifugation at 14,000 *g*, then extracted into Eppendorf tubes and snap‐frozen in liquid N_2_ before storage at −80°C. DNA extraction using 5% Chelex‐100 (Sigma‐Aldrich) resin was performed on red blood cells. PCR was performed as in Fridolfsson and Ellegren (1999), using the primers 2550F (GTTACTGATTCGTCTACGAGA) and 2718R (ATTGAAATGATCCAGTGCTTG), and Go Taq DNA polymerase (Promega). PCR amplicons were visualised in 2% agarose gels. Sex was not determined for 7 chicks due to sample degradation.

### 2.5 Statistical Analyses

All models were fitted with the R (v4.4.1) language and environment (R Core Team 2024).

To examine differences in δ^13^C and δ^15^N within clutches, we used linear mixed‐effects models (LMMs) with a Gaussian error structure. Isotope value (δ^13^C or δ^15^N) was the dependent variable. Position in laying sequence (A, B, C) was included as a fixed effect. Nest ID was incorporated as a random effect to account for non‐independence of sibling eggs. Models were fitted using the lme4 package (Bates et al. 2015). Model assumptions were verified through visual inspection of diagnostic plots.

In addition, egg δ^13^C and δ^15^N repeatability within clutches were assessed using generalised linear mixed‐effects models (GLMMs) to calculate the intraclass correlation coefficient using the package rptR (Stoffel et al. 2017). Nest ID was included as a random effect. To quantify uncertainty in repeatability estimates, we used parametric bootstrapping with 1000 iterations. We assessed repeatability significance using likelihood‐ratio tests, comparing models with and without the random effect. We further validated repeatability estimates using permutation tests (1000), allowing us to quantify the consistency of δ^13^C and δ^15^N values within clutches, accounting for both magnitude of repeatability and estimate uncertainty. This enabled us to infer stable isotope values for unsampled sibling eggs, and to relate this to breeding success.

To investigate parental diet's influence on breeding success, we used linear models, LMMs and GLMMs. Model assumptions were verified through visual inspection of diagnostic plots. First, we investigated lay date using a linear model with lay date of the A‐egg (converted to day of the year, i.e., 1st January = Day 1) as the dependent variable and δ^15^N, δ^13^C, year, and the isotope × year interaction as explanatory variables. The model included 130 eggs; 61 had SIRs measured from sampling and 69 inferred from sibling eggs.

Second, we investigated egg mass using a LMM. Egg mass was the dependent variable, and δ^15^N, δ^13^C, laying sequence, lay date and year were fixed factors. Nest ID was a random factor. The maximal model and simplified versions were equally parsimonious, with ΔAIC < 6 (ΔAIC = 1.62; Richards 2008). Therefore, non‐significant factors were sequentially removed to create a minimal model retaining δ^13^C, egg position, lay date and year as fixed factors. The model included 349 eggs; 154 had SIRs measured from sampling, and 195 inferred from sibling eggs.

Third, we investigated clutch size. Because only six 1‐egg clutches had stable isotope data, and most clutches contained three eggs, we converted clutch size into a binomial variable, with 1‐ and 2‐egg clutches coded as 0 and 3‐egg clutches coded as 1. The model was a binomial GLM. Clutch size was the dependent variable, and δ^15^N, δ^13^C, lay date and year were explanatory variables. For clutches with multiple eggs sampled, one egg was randomly selected to provide data on δ^15^N, δ^13^C and lay date. The maximal model and simplified versions were equally parsimonious (ΔAIC = 3.03). Therefore, non‐significant factors were sequentially removed to create a simplified model. The minimal model retained year as an explanatory variable. It included 137 sampled clutches.

Fourth, we investigated hatching success. Twenty‐four eggs (12 from 2023 and 12 from 2024) which were abandoned, damaged, or preyed on before hatching were discounted from this and further models because they did not reflect parental diet's influence on reproductive success. The model was a binomial GLMM. Hatching success (0 or 1) was the dependent variable, and δ^15^N, δ^13^C, egg position, clutch size, lay date and year were fixed factors. Nest ID was a random factor. Continuous fixed factors were standardised to improve model convergence and interpretability (Schielzeth 2010). The model included 174 eggs.

Fifth, we fitted a LMM with hatchling mass as the dependent variable and δ^15^N, δ^13^C, clutch size, egg position, lay date and year as fixed factors. Nest ID was a random factor. The model was simplified through backward elimination of non‐significant terms to reach a minimal model. All simplified versions were equally parsimonious (ΔAIC = 4.27). The minimal model retained δ^15^N, egg position and year as fixed factors, including 126 chicks.

Finally, we fitted a binomial GLMM, with fledging success of hatched chicks (1 or 0) as the dependent variable. Δ^15^N, δ^13^C, sex, clutch size, egg position, lay date and year were fixed factors. Nest ID was a random factor. Continuous fixed factors were standardised. Because the sample size of the model including sex was only 87 chicks, we removed sex from the model, which did not qualitatively change the result and increased our sample size to 141. We used backwards elimination of non‐significant terms to reach a minimal model with ΔAIC < 6 (ΔAIC = 5.021), retaining δ^15^N and lay date as fixed factors.

We used *R* ^2^ values to assess the variance explained by the models. For mixed‐effects models, we calculated *R* ^2^ values following Nakagawa and Schielzeth (2013). We determined *R* ^2^_GLMM_(m), the marginal *R* ^2^ which represents the variance explained by fixed effects, and *R* ^2^_GLMM_(c), the conditional *R* ^2^, which represents the variance explained by fixed and random effects. Delta *R* ^2^ values are reported for mixed‐effects models because this measures observed variation in the data. *p*‐values less than 0.05 were considered significant. Values are reported as means ± standard errors.

## 3 Results

### 3.1 Within‐Clutch Patterns in Stable Isotopes

**FIGURE 1:**
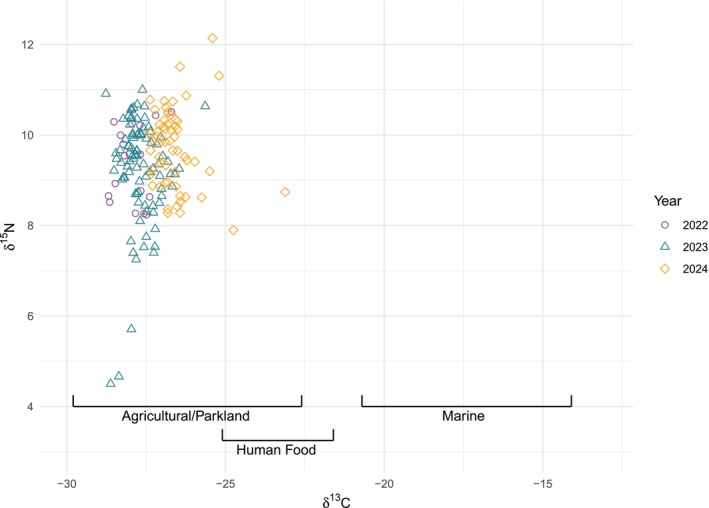
δ^13^C and δ^15^N values from sampled herring gull eggs. Reference ranges of δ^13^C from potential food sources are included, adapted from Swan et al. (2020) (agricultural/parkland), O'Connell and Hedges (1999) and Fletcher et al. (2025) (anthropogenic) and Hobson et al. (1994) and Ronconi et al. (2014) (marine).

The mean δ^13^C in sampled eggs was −27.32 (±0.06), and the mean δ^15^N was 9.41 (±0.08) (Figure 1). Egg position did not significantly affect δ^13^C (linear: *t* = −1.17, *p* = 0.25; quadratic: *t* = −1.01, *p* = 0.32) or δ^15^N (linear: *t* = −1.72, *p* = 0.09; quadratic: *t* = 0.88, *p* = 0.38). Within‐clutch repeatability was high (δ^13^C: *R* = 0.95 ± 0.01, *p* = 2.10 × 10^−14^; δ^15^N: *R* = 0.93 ± 0.02, *p* = 2.08 × 10^−12^). Therefore, for subsequent analyses, we inferred that each egg/chick in the clutch had the same δ^13^C and δ^15^N values as the sampled egg.

### 3.2 Breeding Success Metrics

**FIGURE 2:**
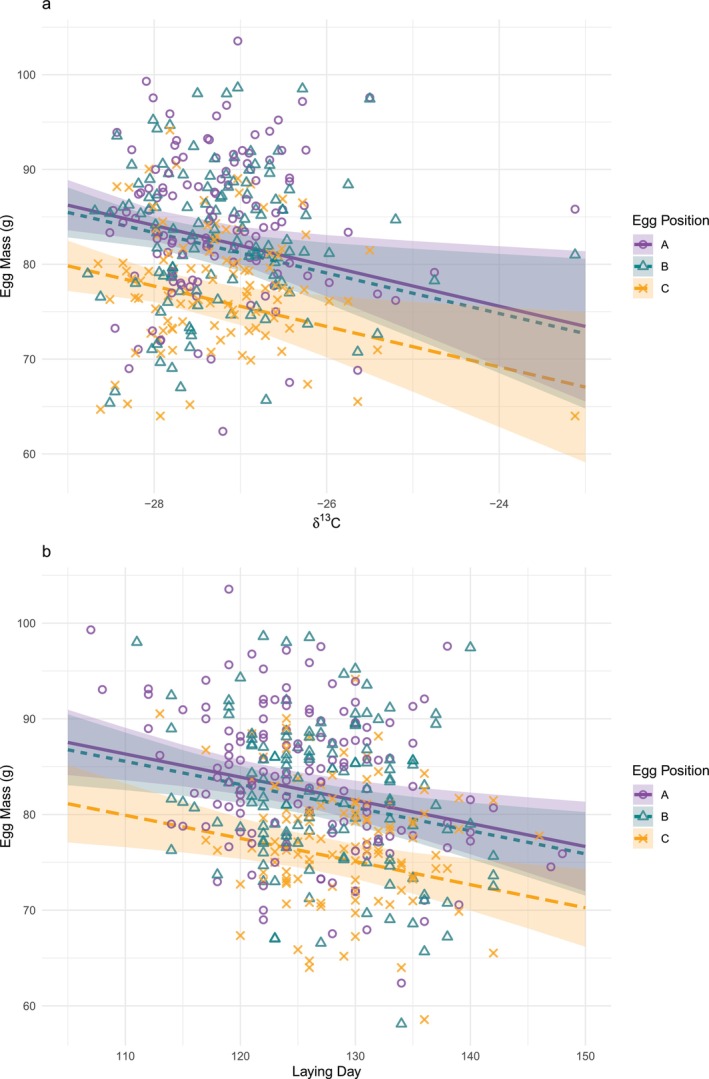
The effect of (a) δ^13^C of egg yolk and lay sequence, and (b) laying day (Day 1 = 1st January) and egg position in the lay sequence, on herring gull egg mass. Regression lines are predicted means for modal year (2023), with shaded areas representing 95% confidence intervals.

Laying date was not significantly related to δ^15^N, δ^13^C, year, or the isotope × year interactions (all *t* < 0.85, *p* > 0.39; *R* ^2^ = 0.14). However, egg mass was significantly negatively associated with δ^13^C and lay date. C‐eggs had significantly lower mass than A‐eggs, and egg mass was significantly lower in 2022 than in 2023 and 2024 (Table 1, Figure 2; *R* ^2^_GLMM_(c) = 0.78, *R* ^2^_GLMM_(m) = 0.27). Gulls were marginally significantly more likely to lay 3‐egg clutches in 2024 (82.5% probability) than in 2022 (50.0%) (Table 2; *R* ^2^ = 0.03).

**TABLE 1:** Outputs from the linear mixed‐effects model with herring gull egg mass (g) as the dependent variable.

Predictor	Coefficient	Std. error	df	*t*	*p*
δ^13^C	−2.13	0.84	133.99	−2.54	0.01
A vs. B‐Egg	−0.77	0.50	266.34	−1.523	0.13
A vs. C‐Egg	−6.40	0.61	338.42	−10.51	1.55 × 10^−22^
Lay date	−0.24	0.08	141.97	−2.93	3.99 × 10^−3^
Year 2022 vs. 2023	6.32	2.76	134.26	2.29	0.02
Year 2022 vs. 2024	10.34	2.91	135.01	3.56	5.19 × 10^−4^

**TABLE 2:** Outputs from the generalised linear model with herring gull clutch size (‘3 eggs’ or ‘< 3 eggs’) as the dependent variable. Coefficients are log likelihoods.

Predictor	Coefficient	Std. error	*z*	*p*
Year 2022 vs. 2023	1.34	0.76	1.75	0.08
Year 2022 vs. 2024	1.55	0.79	1.96	0.05

**FIGURE 3:**
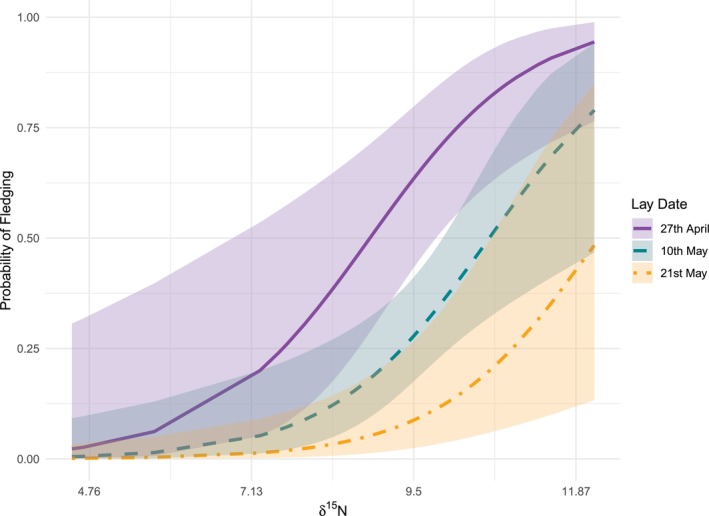
Predicted probability of fledging in herring gull chicks for different δ^15^N values of egg yolk at representative early, middle and late dates in the laying period. Shaded areas represent 95% confidence intervals.

Hatching success was not significantly affected by δ^15^N, δ^13^C, egg position, clutch size, lay date, or year (all *z* < 0.44, *p* > 0.66, *R* ^2^_GLMM_(c) = 1.94 × 10 ^−14^). Excluding eggs that were abandoned, damaged, or preyed on, the overall hatching success rate was 82.8% (169/204). C‐egg hatchlings had significantly lower body mass than A‐egg hatchlings, and hatchlings were significantly heavier in 2024 than in 2023 (Table 3; *R* ^2^_GLMM_(c) = 0.49; *R* ^2^_GLMM_(m) = 0.22). Fledging success was significantly positively associated with δ^15^N and significantly negatively associated with lay date (Table 4, Figure 3; *R* ^2^_GLMM_(c) = 0.44, *R* ^2^_GLMM_(m) = 0.22). Most chick mortality occurred within 5 days post‐hatching (Supporting Information).

**TABLE 3:** Outputs from the GLMM with herring gull Hatchling mass (g) as the dependent variable.

Predictor	Coefficient	Std. error	df	*t*	*p*
δ^15^N	−0.75	0.56	87.76	−1.34	0.18
A vs. B‐Egg	−1.15	1.08	91.98	−1.07	0.29
A vs. C‐Egg	−4.86	1.02	84.09	−4.78	7.29 × 10^−6^
Year (2024)	2.82	1.08	83.20	2.62	0.01

**TABLE 4:** Outputs from the GLMM with fledging success as the dependent variable. Coefficients are log likelihoods.

Predictor	Coefficient	Std. error	*z*	*p*
δ^15^N	1.02	0.39	2.64	0.01
Lay date	−0.78	0.33	−2.34	0.02

## 4 Discussion

This study reports a strongly terrestrial stable isotope signature in the eggs of herring gulls breeding in Cornwall, UK, indicating feeding primarily in agricultural and parkland habitats rather than on human food or refuse. Lower δ^13^C values (indicative of more terrestrial diets) were associated with higher egg mass. Fledging success correlated positively with maternal trophic level (indicated by higher δ^15^N values). Although hatching success was not associated with maternal diet, most chick mortality occurred within 5 days post‐hatching. This suggests a lagged impact of hatching stress, potentially influenced by egg nutrition. Newly hatched chicks are vulnerable to oxidative stress caused by the exertion of hatching and sudden exposure to atmospheric oxygen concentrations (Freeman and Vince 1974; Surai et al. 1996).

As predicted, we found no variation over the laying sequence, and high repeatability in δ^13^C and δ^15^N values among sibling eggs. This agrees with a study on egg stable isotopes in European starlings ( *Sturnus vulgaris* ) (Yohannes et al. 2016). Thus, we can infer maternal diet during egg formation from stable isotope data from a single egg and relate this to sibling eggs and chicks' development and survival. Δ^13^C values from eggs (Figure 1) align with δ^13^C values in terrestrial ecosystems, which are ^13^C‐depleted compared to marine ecosystems (Peterson and Fry 1987; Hobson et al. 1994; Knoff et al. 2002; Ronconi et al. 2014). The δ^13^C isotopic signature largely matches natural terrestrial diets rather than anthropogenic food (O'Connell and Hedges 1999; Hopkins and Ferguson 2012; Shlepr et al. 2021; Fletcher et al. 2025), This indicates that, in this colony, resources for egg formation are primarily from feeding on agricultural and park land, with minimal marine or anthropogenic components. Traditional methods of quantifying nutrient contributions based on relative tissue mass indicate that exogenous and endogenous nutrient proportions can vary in *Larus* gull eggs, although exogenous nutrients play a key role (Houston et al. 1983; Hario et al. 1991). Studies of SIRs in gull eggs found that nutrient investment was primarily exogenous and likely reflected local food availability (Hobson et al. 1997, 2000). Supplemental feeding of gulls with carotenoids or protein during pre‐laying improved breeding success (Bolton et al. 1992; Blount et al. 2002, 2004). Studies of amino acid‐specific SIRs further support a strong exogenous component to nutrient allocation in herring gull eggs (Hebert et al. 2016; Whiteman et al. 2021). Even where nutrient investment is endogenous, it may still come from sources close to the breeding colony because several studies link increased tissue protein and lipid stores of pre‐breeding female gulls to male courtship feeding before laying (Norstrom et al. 1986; Hario et al. 1991; Mawhinney et al. 1999). Consequently, this strongly terrestrial isotope signature likely indicates diet just before and during egg formation.

Local resource availability is a key factor in dietary preferences during nesting. Breeding herring gulls in Newfoundland exhibited dietary specialisation based on nesting habitat, driven by the opportunity costs of foraging, such as the risk of nest predation or nest site theft while parents were foraging. In habitats where these risks were greater, gulls were more likely to specialise in food that was closer to the nest and took less time to obtain (Pierotti and Annett 1991). Herring gulls in the Great Lakes showed considerable regional diet variation, with gulls near farmland preferring small mammals, whereas others focused on fish (Ewins et al. 1994). O'Hanlon et al. (2017) found breeding herring gulls in Scotland and Northern Ireland fed on more terrestrial resources near urban areas and more intertidal resources along sheltered coastlines. Urban and coastal lesser black‐backed gulls preferentially foraged in their nesting habitat (Langley et al. 2023). Yellow‐legged gulls ( *Larus michahellis* ) reduced marine prey consumption with increasing distance from fishing harbours (Zorrozua et al. 2020). Gyimesi et al. (2016) reported an inland colony of lesser black‐backed gulls feeding entirely terrestrially, with high breeding success. The strong preference for natural, terrestrial food in our study colony likely reflects local resource availability, although it is surprising because the colony is semi‐urban and < 2 km from the coast. The average foraging range of breeding herring gulls is 10.5 km (Thaxter et al. 2012), which here encompasses urban, rural, marine and estuarine habitats. Even with multiple available options, food may be most abundant and accessible in agricultural habitats for this colony. Some studies show terrestrial foraging is preferred during pre‐laying, with a shift to marine foraging while provisioning chicks (Annett and Pierotti 1989; Perrins and Smith 2000; Isaksson et al. 2016). The reverse was seen in urban gulls in New York (Washburn et al. 2013), so a switch from terrestrial to marine foraging over the breeding period is not universal and likely depends on resource availability and quality. The terrestrial foraging preference here may indicate that nearby marine habitat offers poor profitability, warranting further study.

Alternatively, the dietary preference we observed may indicate adaptive decisions to sequester an optimal nutrient balance for egg production. Sulphur amino acids and antioxidants can be limiting for egg production (e.g., Murphy 1994; Blount et al. 2004) and several passerine species demonstrate dietary discrimination for these nutrients (Murphy and King 1987; Schaefer et al. 2008; Beaulieu and Schaefer 2014). The significantly increased egg mass at lower values of δ^13^C supports this conclusion. However, this contradicts prior studies showing that marine and intertidal foraging improve breeding success in *Larus* gulls (Annett and Pierotti 1999; O'Hanlon et al. 2017). This may indicate poor foraging profitability in nearby marine habitats. Alternatively, marine‐feeding gulls may expend more energy in foraging due to greater travel distances, leaving fewer resources for eggs. Higher δ^13^C may also indicate increased anthropogenic feeding rather than marine feeding, resulting in lower egg mass due to poorer nutritional quality of human refuse. Further investigation through faecal metabarcoding and tracking studies is needed to clarify the dietary preference in this colony and to confirm whether a switch to marine feeding occurs later during breeding.

Our study showed a positive relationship between fledging success and egg δ^15^N, even when accounting for decreased fledging success with later laying (Brouwer et al. 1995; Bukacińska et al. 1996). Higher values of δ^15^N are associated with higher trophic levels (Rau 1982; Hobson et al. 1994; Kelly 2000). Therefore, gulls feeding at higher trophic levels in early reproduction have elevated breeding success. The same pattern was not seen for hatching success, nor for egg and hatchling masses. However, high chick mortality within 5 days post‐hatching suggests egg phenotype or hatching stress influenced chick survival. Cross‐fostering experiments will help clarify the effects of maternal dietary trophic level on chick fledging success.

Comparison of SIRs from our study to data for buzzard ( *Buteo buteo* ) diet in the same locality (Swan et al. 2020) suggests gulls were primarily feeding on small herbivorous mammals, such as young rabbits (*Oryctolagus caniculus*) and rodents, which are important prey for larids, especially in inland colonies or during marine food shortages (Camphuysen et al. 2010). Gulls in the study area have been observed eating rats ( *Rattus norvegicus* ) (pers. obs.). The highest δ^15^N values observed are consistent with a diet of small carnivorous mammals such as shrews (*Sorex* spp.); shrew jaw bones have been found in regurgitated pellets at this colony (pers. obs.). Lower δ^15^N values may indicate a diet containing more invertebrates. Observations during chick rearing showed abundant chafer beetles (Scarabaeidae) in pellets, and earthworms (Lumbricidae) are an important terrestrial food source for herring gulls (Götmark 1984; Perrins and Smith 2000). Investigation of dietary contents using faecal metabarcoding will be invaluable in identifying gulls' dietary preferences.

Other studies have investigated relationships between trophic level and fitness in birds. Ronconi et al. (2014) demonstrated a link between higher trophic level and improved body condition in herring gulls, supporting our findings. However, in Arctic seabirds, including glaucous gulls ( *Larus hyperboreus* ), feeding at higher trophic levels in the non‐breeding season was consistently associated with lower hatching success the following year over 7 years of study (Hovinen et al. 2019). As the authors speculate, this association may be driven by fluctuations in food availability and productivity, with lower trophic‐level organisms representing a larger proportion of both overall biomass and seabird diet in high productivity years (and vice‐versa). Alternatively, this may be a result of Arctic birds using more endogenous resources in low‐productivity years. Both of these explanations may explain the contradiction between the current study and that of Hovinen et al. (2019). In aquatic systems, feeding at higher trophic levels may increase exposure to contaminants like heavy metals (Saidon et al. 2024) or phthalates (Allen et al. 2021), countering nutritional benefits. In marbled murrelets ( *Brachyramphus marmoratus* ), birds fed on the most abundant prey each year, altering trophic level accordingly, but breeding success was linked most strongly to mid‐trophic level prey consumption (Becker et al. 2007). Urban‐nesting blue tits ( *Cyanistes caeruleus* ) fed at higher trophic levels than suburban‐ and forest‐nesting populations, with lower hatching and fledging success due to shifting away from caterpillars as nestling food (Pollock et al. 2017). Clearly, trophic level and breeding success can be coupled or decoupled depending on birds' feeding ecology, nutritional requirements and prey availability. In our system, high‐trophic level terrestrial prey may best meet gulls' nutritional requirements during egg formation or optimise the trade‐off between nutrition and foraging time.

## 5 Conclusion

Our findings demonstrate that δ^13^C and δ^15^N values are consistent within clutches, allowing reliable inference of SIRs in siblings from sampling of a single egg. Our data indicate a strong preference for agricultural/parkland foraging for egg production in this colony, show an increase in egg mass with more terrestrial foraging, and show that feeding at higher trophic levels is associated with improved fledging success. These results suggest that, for this colony, a preference for high trophic level terrestrial prey during pre‐laying and laying may be adaptive. Comparison with known SIRs of possible prey items and gull dietary preferences indicates that small mammals are likely the main dietary component. Future cross‐fostering experiments may clarify whether improved fledging success associated with higher trophic level foraging is a function of nutritional investment in eggs or parental condition associated with diet.

The strong preference for terrestrial food sources in this herring gull colony is surprising in a pericoastal, roof‐nesting population, highlighting the variable foraging strategies of this species. Studying the feeding preferences of urban wildlife such as herring gulls is key to understanding how species adapt to urbanised environments. Maternal diet and effects on egg quality are likely key determinants of breeding success in gulls. Understanding breeding success in UK herring gulls is important due to their nationally Red‐Listed status and internationally important numbers. Herring gulls are generalist foragers typically considered to increasingly rely on anthropogenic food resources (Pons and Migot 1995; Steigerwald et al. 2015; Gyimesi et al. 2016; Goumas et al. 2020). Further research into the diets of individuals breeding in various ecological contexts may provide crucial insights into how animal species such as gulls can exploit human‐modified environments.

## Author Contributions

**Simon F. Allen:** data curation (equal), formal analysis (lead), investigation (supporting), writing – original draft (lead). **Richard Inger:** conceptualization (supporting), data curation (equal), formal analysis (supporting), investigation (equal), methodology (equal), supervision (supporting), writing – original draft (supporting). **Paige E. Petts:** investigation (equal). **Jody M. Affleck:** investigation (equal). **Katie Bennett:** investigation (equal). **Tom W. Davies:** investigation (equal). **Ben D. Haden:** investigation (equal). **Luke C. Hughes:** investigation (equal). **Neeltje J. Boogert:** investigation (equal). **Chris Mitchell:** data curation (equal), investigation (equal), methodology (equal). **Eva Jimenez‐Guri:** data curation (equal), investigation (equal), methodology (equal). **Jonathan D. Blount:** conceptualization (lead), funding acquisition (lead), investigation (equal), methodology (equal), project administration (lead), resources (lead), supervision (lead), writing – original draft (supporting), writing – review and editing (lead).

## Conflicts of Interest

The authors declare no conflicts of interest.

## Supporting information


**Appendix S1:** ece372307‐sup‐0001‐AppendixS1.docx.

## Data Availability

Data and code are available at https://doi.org/10.5061/dryad.05qfttfg4.
