# Supplementary material for: Predominantly Terrestrial Foraging and Reproductive Gains From a High Trophic Level Diet in Roof‐Nesting Herring Gulls (Larus argentatus)
Source: Ecol Evol. 2025 Oct 10;15(10):e72307. doi: 10.1002/ece3.72307 (PMC12513736; doi:10.1002/ece3.72307)
Supplement: Supplementary file 1 — Appendix S1: ece372307‐sup‐0001‐AppendixS1.docx. [file ECE3-15-e72307-s001.docx]

**Predominantly terrestrial foraging and reproductive gains from a high trophic level diet in roof-nesting herring gulls (*Larus argentatus*) – Supplemental Material**

| **Fate of Egg** | **2022** | **2023** | **2024** |
| --- | --- | --- | --- |
| Sampled | 18 | 92 | 57 |
| Egg Failed | N/A | 39 | 36 |
| Chick Died | N/A | 59 | 42 |
| Chick Fledged | N/A | 38 | 33 |

**Table 1.** General fates of all herring gull eggs for the years 2022, 2023, and 2024.


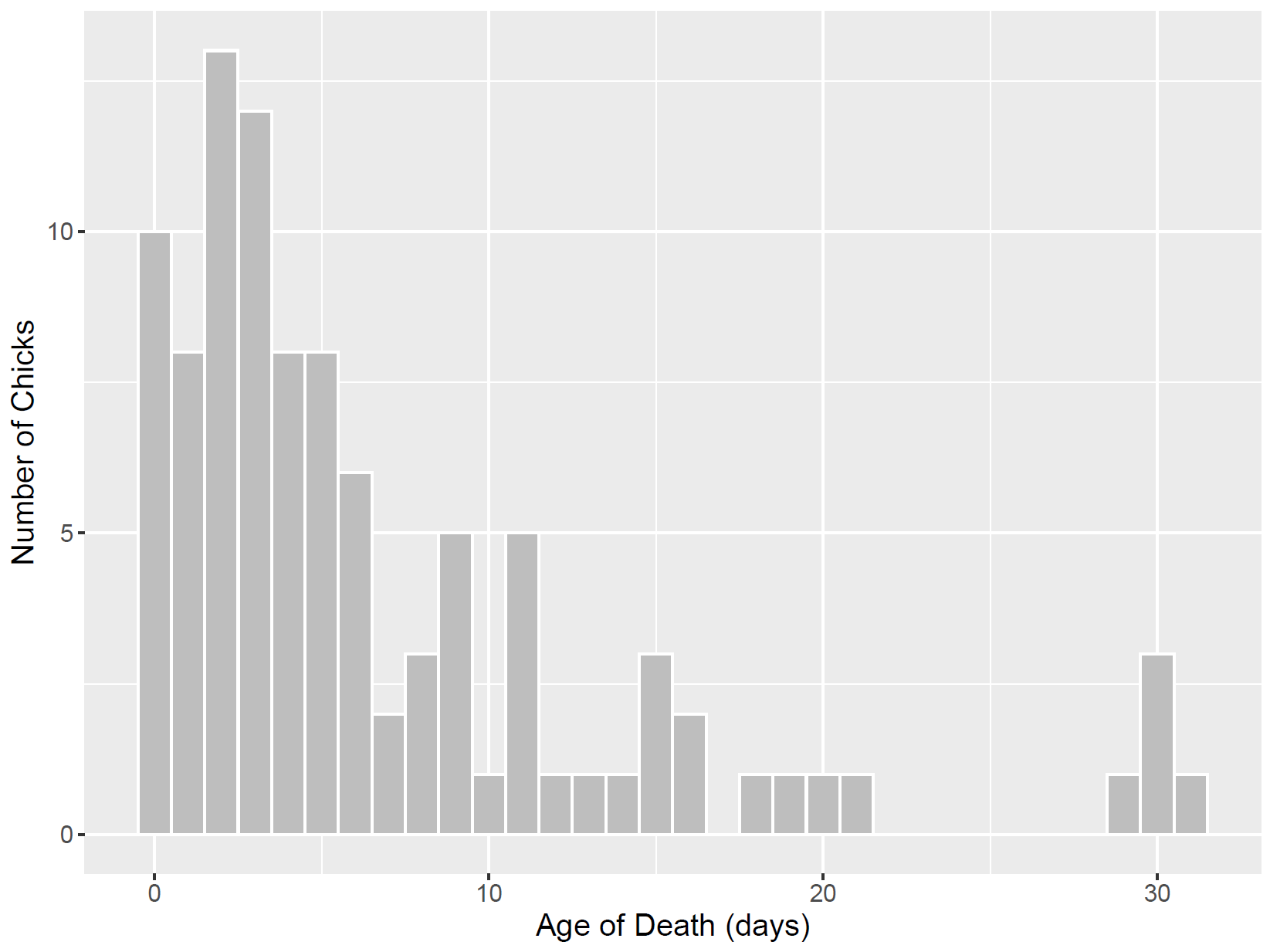


**Figure 1.** Age of death in herring gull chicks which hatched but failed to fledge throughout the years 2023 and 2024.
